# Multi-system trajectories and the incidence of heart failure in the Framingham Offspring Study

**DOI:** 10.1371/journal.pone.0268576

**Published:** 2022-05-26

**Authors:** Cara E. Guardino, Stephanie Pan, Ramachandran S. Vasan, Vanessa Xanthakis

**Affiliations:** 1 Division of Cardiology, Warren Alpert Medical School of Brown University and Lifespan Cardiovascular Institute, Providence, Rhode Island, United States of America; 2 Department of Medicine, Boston University School of Medicine and Boston Medical Center, Boston, Massachusetts, United States of America; 3 Department of Biostatistics, Boston University School of Public Health, Boston, Massachusetts, United States of America; 4 National Heart, Lung, and Blood Institute’s and Boston University’s Framingham Heart Study, Framingham, Massachusetts, United States of America; 5 Section of Preventive Medicine and Epidemiology, Boston University School of Medicine, Boston, Massachusetts, United States of America; 6 Section of Cardiology, Boston University School of Medicine, Boston, Massachusetts, United States of America; 7 Department of Epidemiology, Boston University School of Public Health, Boston, Massachusetts, United States of America; University of Alberta, CANADA

## Abstract

**Background:**

Heart failure is a multi-system disease, with non-cardiac systems playing a key role in disease pathogenesis.

**Objective:**

Investigate whether longitudinal multi-system trajectories incrementally predict heart failure risk compared to single-occasion traits.

**Methods:**

We evaluated 3,412 participants from the Framingham Heart Study Offspring cohort, free of heart failure, who attended examination cycle 5 and at least one examination between 1995–2008 (mean age 67 years, 54% women). We related trajectories for the following organ systems and metabolic functions to heart failure risk using Cox regression: kidney (estimated glomerular filtration rate), lung (forced vital capacity and the ratio of forced expiratory volume in one second/forced vital capacity), neuromotor (gait time), muscular (grip strength), cardiac (left ventricular mass index and heart rate), vascular function (pulse pressure), cholesterol (ratio of total/high-density lipoprotein), adiposity (body mass index), inflammation (C-reactive protein) and glucose homeostasis (hemoglobin A1c). Using traits selected via forward selection, we derived a trajectory risk score and related it to heart failure risk.

**Results:**

We observed 276 heart failure events during a median follow up of 10 years. Participants with the ‘worst’ multi-system trajectory profile had the highest heart failure risk. A one-unit increase in the trajectory risk score was associated with a 2.72-fold increase in heart failure risk (95% CI 2.21–3.34; p<0.001). The mean c-statistics for models including the trajectory risk score and single-occasion traits were 0.87 (95% CI 0.83–0.91) and 0.83 (95% CI 0.80–0.86), respectively.

**Conclusion:**

Incorporating multi-system trajectories reflective of the aging process may add incremental information to heart failure risk assessment when compared to using single-occasion traits.

## Introduction

The prevalence of heart failure (HF) among adults 20 years of age and older in the United States is estimated to be 6.2 million based on data accrued from the National Health and Nutrition Examination Survey between 2013–2016 [1]. From 2012–2030, the prevalence of HF is expected to increase by 46% [1]. Despite advancements in treatment, the diagnosis of HF continues to portend an extremely poor prognosis [1–3]. Given the high mortality rate once the diagnosis is made, a better understanding of the clinical syndrome before the manifestation of the disease is imperative. The increasing incidence of HF has been attributed to the aging population [3]. However, it remains unclear why the extent of cardiac dysfunction, a potential surrogate of cardiac aging, does not consistently correlate with the clinical severity of the condition (in terms of symptoms); a potential explanation could be the fact that HF is a multi-system syndrome with several non-cardiac predictors [4].

Prior studies have focused on the relation between a set of traits measured at one point in time and the risk of HF [4–24]. Each organ system follows a different trajectory of aging and decline, which may have a variable impact on HF risk [25–27]. However, to our knowledge, data are lacking on whether the decline of the function of multiple organ systems over time is associated with HF risk. Moreover, it is not clear whether the trajectories of the function of various organ systems conjointly could provide better discrimination ability with regards to HF risk compared to single-occasion measurements of the same organ systems.

In this investigation, we hypothesized that the distinct long-term trajectory patterns of multiple organ systems are individually related to HF risk. Moreover, we postulated that collective-trajectory profiles of key organ systems might provide better discrimination information in terms of HF risk compared to single-occasion measurements. We evaluated these hypotheses using data from the Framingham Heart Study (FHS) Offspring cohort. We chose 12 distinct organ systems, previously related to cardiovascular disease (CVD), CVD mortality, and all-cause mortality, as a comprehensive representation of the body’s aging [25].

## Methods

Data for the Framingham Offspring Study examinations can be obtained from the National Heart, Lung, and Blood Institute’s data repository at https://biolincc.nhlbi.nih.gov/studies/.

### Study sample

Prior investigations have published on the inclusion and exclusion criteria of the FHS cohorts and its study design [28]. Of the 3,799 Offspring cohort participants who attended the fifth examination cycle (1991–1995), we excluded 250 participants who did not attend at least one other examination after the fifth, i.e., the sixth (1995–1998), seventh (1998–2001), or eighth (2005–2008) examination cycles. Further, we excluded participants with prevalent HF at their most recent examination attendance (n = 111), had missing baseline covariates (n = 11), or had no follow-up information (n = 15), resulting in a final sample size of 3,412 participants. We applied the eligibility criteria at the start of the fifth examination cycle since the purpose of this investigation was to examine mid- to late-life biological system trajectories in relation to HF risk, and serial measurements for the selected traits were available starting at the fifth examination cycle.

All participants provided written informed consent. Approval for this investigation was obtained from the Institutional Review Board at the Boston University Medical Center.

### Organ system traits

For the present investigation, we used the following traits and their corresponding representative body organ system: estimated glomerular filtration rate, eGFR (kidney function); forced vital capacity, FVC, and the ratio of forced expiratory volume in one second and FVC, FEV1/FVC (pulmonary function); gait time (neuromotor function); grip strength (muscular function); left ventricular mass indexed by height, LVMI and heart rate, HR (cardiac function); pulse pressure, PP (vascular function); the ratio of total cholesterol and high-density lipoprotein cholesterol, TC/HDL (cholesterol metabolism); C-reactive protein, CRP (inflammation); body mass index, BMI (adiposity); and hemoglobin A1c, HbA1c (glucose homeostasis).

We selected these unique traits because they have previously been associated with CVD, CVD-related death, and all-cause mortality [25]. These traits provide a comprehensive representation of multiple organ systems and the metabolism of individuals [25].

### Outcome of interest

The primary outcome of this investigation was incident HF. Participants were followed for the development of HF from their most recent examination cycle attendance (sixth, seventh or eighth) through December 31, 2017. Surveillance of the participants for HF outcomes included collecting their health history, reviewing FHS visits and medical records from outpatient visits, and hospitalizations related to HF. An adjudication panel consisting of three FHS investigators evaluated all outcomes. The diagnosis of HF was based on the previously established Framingham criteria for congestive HF requiring the presence of two major criteria or one major and two minor criteria [29].

## Statistical analysis

Descriptive statistics for the sample characteristics were reported from the most recent examination attended by the participants.

For each trait, we created two exposure variables: one representing a single occasion measurement and another representing the trajectory-based longitudinal pattern. The most recent measurements collected between 1991 and 2008 were used as the single time point exposures.

We estimated sex-specific age-adjusted Spearman partial correlation coefficients among the single-occasion traits.

### Association of single-occasion traits with HF risk

After confirming that the proportional hazards assumption was met, we estimated Cox proportional hazards regression models to relate each single-occasion trait to the time to incident HF (separate model for each of the 12 traits). Models were adjusted for age, sex, smoking, BMI (or weight for traits indexed by height), antihypertensive treatment, diabetes, TC/HDL, and SBP (except when evaluating PP to avoid multicollinearity). The most recent measurements collected between 1991 and 2008 were used as the single time point exposures.

### Group-based trajectories of traits

We created group-based model trajectories for each of the 12 traits, using at least two measurements for each trait from each participant (one from the fifth examination cycle and all other available measurements from exams six through eight). Group-based trajectory modeling assumes unique subgroups within the sample, each with its underlying trajectory. We used SAS Proc Traj, a finite mixture model procedure using maximum likelihood, to identify groups of participants who follow a similar progression of traits [30]. In determining the group-based trajectory model that best fits each trait, we followed an algorithm proposed by Jones and Nagin [31, 32]. We decided *a priori* to create models ranging from one to five groups with each set to a quadratic order for comparison purposes. All traits were continuous and assumed to have a censored normal distribution. Using the Bayesian Information Criterion (BIC), we compared the goodness-of-fit between the models in a stepwise manner to establish our decision on the number of groups with the aim to have the estimated membership in each group be at least 5%. Next, we fitted the model by varying the polynomial degrees (i.e., quartic, cubic, quadratic, and linear) to determine the shape of each group’s trajectory over time. The final fitted model computes an individual’s posterior probability of group membership and assigns the person to the group with the highest posterior probability. To confirm that our model adequately fit the data, we performed a diagnostic assessment by verifying that the average posterior probabilities of the group an individual was assigned to exceeded 0.7. Once we determined the best model fit and confirmed the model diagnostics for each trait, each participant was classified into a trajectory group based on their estimated highest posterior probability of group membership. CRP was winsorized at the 95^th^ percentile by exam and sex, and was then log-transformed before model fitting. In fitting the group-based trajectory models, all traits were adjusted for age at examination cycle five with the assumption that it is a risk factor affecting the probability of group membership. Depending on the trait, we further included a time-varying covariate in estimating the trajectory groups: height (for models including FEV1/FVC and FVC), diabetes medication use (for HbA1c), beta-blocker use (for HR), and lipid-lowering medication use (for TC/HDL). As an exploratory analysis, we excluded participants with prevalent MI and/or previous cardiac surgery when creating the group-based trajectories. Moreover, we also performed sex-specific exploratory analysis of the single-occasion and group trajectories association in relation to HF risk (with corresponding sex-specific trajectory profiles).

### Association of group-based trajectories with HF risk

After estimating the trajectory profile group for each trait using group-based modelling, we created binary variables for traits resulting in two distinct trajectory profiles (1 = ‘best’ and 2 = ‘worst’) and 3-level variables for traits resulting in three distinct trajectory profiles (1 = ‘best’, 2 = ‘intermediate’, and 3 = ‘worst’). We related the derived variables (‘best’ serving as the referent group) to HF risk using Cox proportional hazards regression models (separate model for each trait): (1) adjusting for age and sex and (2) adjusting for age, sex, smoking status, BMI (or weight for traits indexed by height), antihypertensive treatment, diabetes status, TC/HDL, and SBP (except when evaluating PP to preclude multicollinearity). The proportional hazards assumption was met for all models. Follow up for the development of HF started after the most recent examination for each participant.

### Model discrimination for single-occasion models vs. group-based trajectory models

We compared the discrimination ability of models including single-occasion variables versus those including group-based trajectory variables for each trait, using 500 bootstrapped samples by estimating the mean Harrell’s c-statistic index (with 95% CI). In each bootstrap, a c-statistic index was obtained for three Cox proportional hazards models: (1) covariates-only model (S_0_), (2) covariates plus the single-occasion variable (S_1_), and (3) covariates plus the group-based trajectory variable (S_2_). In addition, we computed the mean change in c-statistic between a model with only covariates and a model including covariates plus the single-occasion variable (ΔS_1_ = S1 − S_0_). We also calculated the mean change in c-statistic between a model with only covariates and a model including covariates plus the group-based trajectory variable (ΔS_2_ = S2 − S_0_). The set of covariates (i.e., age, sex, smoking, BMI, etc.) in these models were consistent with the prior models. The bootstrap process was performed using the SAS macro %boot.

### Derivation of the trajectory risk score

We used a forward selection algorithm in a Cox proportional hazards model that incorporated all traits and selected variables using the likelihood ratio test. Multicollinearity was inspected among the group-based trajectory variables prior to the forward selection process. The proportional hazards assumption was evaluated and confirmed at each step of the variable selection. We then created a composite trajectory risk score using the variables that were selected from the forward algorithm as follows: we calculated the sum of the scores previously assigned to the trajectory variables (1 = ‘best,’ 2 = ‘intermediate,’ 3 = ‘worst’), weighted by the corresponding regression coefficients. Then, we related the derived composite trajectory risk score to HF risk using a Cox proportional hazards model, adjusted for age and sex. Finally, we created tertiles of the composite trajectory risk score and created unadjusted cumulative incidence curves.

### Model discrimination for multiple single-occasion traits model vs. trajectory risk score model

To compare the discrimination ability of a model including the trajectory risk score to a model including multiple single-occasion variables, we used a process similar to what was described above for discriminating single-occasion models and group-based trajectory models. The c-statistic index obtained for the three Cox proportional hazards models include: (1) age-sex only model (M_0_); (2) age, sex, plus trajectory risk score (M_1_); and (3) age, sex, plus multiple single-occasion variables (M_2_). The traits selected for the multiple single-occasion model are based on the aforementioned forward selection process. As described above, we calculated the mean change in c-statistic from the age- and sex-adjusted model to the model, including age, sex, and the trajectory risk score (ΔM_1_ = M1 − M_0_), as well as the mean change in c-statistic from the age- sex-adjusted model to the model, including age, sex, and the multiple single-occasion variables (ΔM_2_ = M2 − M_0_).

Statistical significance was evaluated using a two-sided P<0.05 unless otherwise stated. All analyses were performed using SAS version 9.4 (Cary, NC).

## Results

### Study sample

The characteristics of our study sample, stratified by sex, are shown in Table 1. Our sample had an average age of 67 years, and slightly over half were women (54%). On average, participants were overweight, and approximately half were on antihypertensive treatment. Details on the average and maximum number of measurements per participant for each trait are included in S3 Table.

**Table 1:** Baseline characteristics of study sample, stratified by sex.

Characteristics	Men (n = 1573)	Women (n = 1839)
Age, years	66.5 ± 9.2	66.7 ± 9.6
Height, meters	1.7 ± 0.1	1.6 ± 0.1
Weight, kg	87.7 ± 15.9	71.3 ± 15.9
Smoking, n (%)	162 (10.3)	201 (10.9)
Diabetes, n (%)	280 (17.8)	209 (11.4)
Lipid Treatment, n (%)	694 (44.1)	629 (34.2)
Systolic Blood Pressure, mmHg	129.9 ± 17.3	129.1 ± 18.4
Diastolic Blood Pressure, mmHg	75 ± 10	73 ± 10
Antihypertensive Treatment, n (%)	811 (51.6)	849 (46.2)
Traits		
eGFR, mL/min	79 ± 17	78 ± 17
Hemoglobin A1c, %	5.8 ± 1	5.7 ± 0.7
Body Mass Index, kg/m^2^	28.8 ± 4.7	27.8 ± 5.9
Pulse Pressure, mmHg	58 ± 17	59 ± 18
C-Reactive Protein, mg/L	1.6 (0.8, 3.4)	1.8 (0.9, 4.2)
Heart Rate, beats per minute	62 ± 11	65 ± 11
Total/HDL Cholesterol, mg/dL	4.0 ± 1.8	3.4 ± 1.1
FVC, mL	4298 ± 903	3009 ± 609
FEV1/FVC, %	71 ± 9	72 ± 8
Left Ventricular Mass Index, g/m^2^	115 ± 26	90 ± 20
Gait Time, s	3.5 ± 0.9	3.6 ± 1.0
Grip Strength, kg	41 ± 10	23 ± 6

Data are shown as mean ± SD or median (quartile 1, quartile 3) for continuous variables and frequency (%) for categorical variables. Left ventricular mass is indexed to height. Sample characteristics are based on the most recent examination attended by the participants.

eGFR = estimated glomerular filtration rate; FEV1 = forced expiratory volume; FVC = forced vital capacity; HDL = high-density lipoprotein.

### Correlation among traits

**Fig 1:**
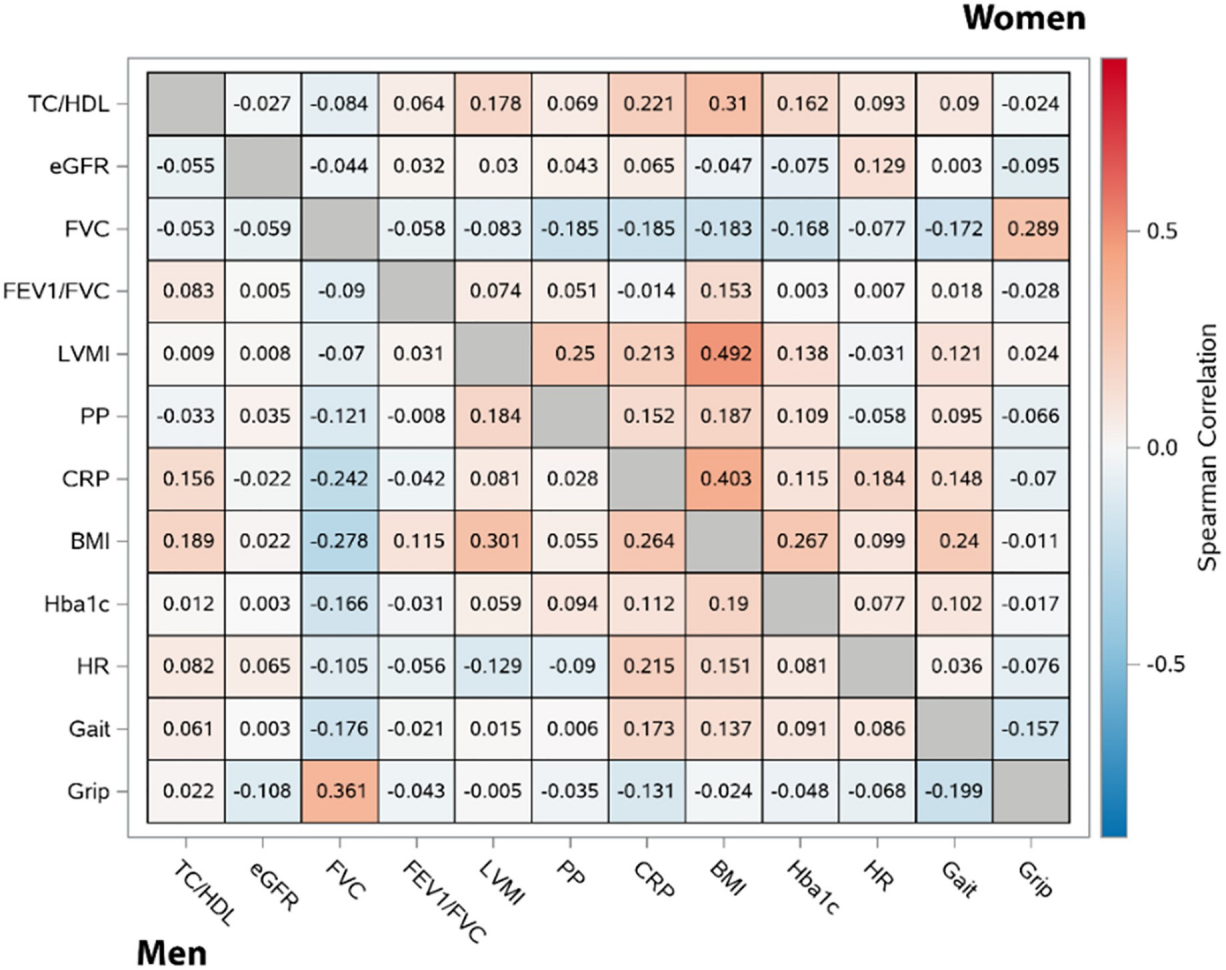
Sex-specific spearman correlations, adjusted for age. Correlations among single-occasion traits using Spearman partial correlation coefficients, adjusting for age, are depicted. Men (n = 1179) are shown in the lower triangle, and women (n = 1456) are shown in the upper triangle. Deeper red = stronger positive correlation; deeper blue = stronger negative correlation. BMI = body mass index; CRP = C-reactive protein; eGFR = estimated glomerular filtration rate; FEV1 = forced expiratory volume; FVC = forced vital capacity; HbA1c = hemoglobin A1c; HDL = high-density lipoprotein; HR = heart rate; LVMI = left ventricular mass index; PP = pulse pressure; TC = total cholesterol.

Age-adjusted and sex-stratified Spearman correlations among the 12 surrogate traits are illustrated in Fig 1. In men, we observed moderate correlations of FVC with grip strength, and of BMI with LVMI (Fig 1). In women, there were also modest correlations of BMI with TC/HDL, LVMI, and CRP.

### Association of single-occasion traits with HF risk

We observed 276 new-onset HF events over a median follow-up period of 10.3 years. Adjusting for age, sex, smoking status, BMI (or weight for traits indexed by height), antihypertensive treatment, diabetes status, TC/HDL, and SBP (except when evaluating PP), we observed positive associations of HbA1c, BMI, PP, CRP, HR, TC/HDL, LVMI, and gait with HF risk. We also observed an inverse association of eGFR and FVC with HF risk. (Table 2)

**Table 2:** Associations of single-occasion traits and group trajectories with HF risk.

Trait	Model 1 –Single Occasion			Model 2 –Group Trajectory		
	# events/# at risk (%)	HR (95% CI)	p-value*	# events/# at risk (%)	HR (95% CI)	p-value*
**eGFR**	274/3401 (8.1)	0.88 (0.77, 1.00)	**0.049**	262/3312 (7.9)		0.08
Best				34/1128 (3)	Reference	--
Intermediate				142/1692 (8.4)	1.20 (0.79, 1.83)	0.38
Worst				86/492 (17.5)	1.61 (0.99, 2.63)	0.06
**HbA1c**	262/3319 (7.9)	1.25 (1.12, 1.39)	**< .0001**	205/2794 (7.3)		**0.02**
Best				184/2689 (6.8)	Reference	--
Worst				21/105 (20)	1.86 (1.12, 3.08)	0.02
**BMI**	276/3412 (8.1)	1.38 (1.23, 1.55)	**< .0001**	270/3359 (8)		**< .0001**
Best				101/1692 (6)	Reference	--
Intermediate				129/1395 (9.3)	1.26 (0.96, 1.66)	0.09
Worst				40/272 (14.7)	2.48 (1.67, 3.68)	< .0001
**PP**	276/3412 (8.1)	1.16 (1.02, 1.32)	**0.03**	276/3412 (8.1)		**0.0001**
Best				52/1740 (3)	Reference	--
Intermediate				139/1271 (10.9)	1.66 (1.14, 2.40)	0.008
Worst				85/401 (21.2)	2.57 (1.65, 4.01)	< .0001
**CRP**	269/3350 (8)	1.26 (1.14, 1.41)	**< .0001**	229/3052 (7.5)		**0.0003**
Best				14/563 (2.5)	Reference	---
Intermediate				95/1459 (6.5)	1.63 (0.93, 2.88)	0.09
Worst				120/1030 (11.7)	2.62 (1.47, 4.66)	0.001
**HR**	276/3412 (8.1)	1.20 (1.07, 1.35)	**0.0015**	276/3412 (8.1)		**0.0001**
Best				60/1127 (5.3)	Reference	--
Intermediate				148/1830 (8.1)	1.44 (1.06, 1.95)	0.02
Worst				68/455 (15)	2.23 (1.54, 3.22)	< .0001
**TC/HDL**	276/3412 (8.1)	1.12 (1.05, 1.20)	**0.0005**	269/3350 (8)		0.34
Best				163/2268 (7.2)	Reference	--
Worst				106/1082 (9.8)	1.13 (0.88, 1.46)	0.34
**FVC**	236/3133 (7.5)	0.55 (0.45, 0.69)	**< .0001**	224/3041 (7.4)		**< .0001**
Best				13/628 (2.1)	Reference	--
Intermediate				82/1512 (5.4)	1.79 (0.99, 3.25)	0.05
Worst				129/901 (14.3)	2.99 (1.64, 5.45)	0.0003
**FEV1/FVC**	259/3299 (7.9)	0.91 (0.80, 1.03)	0.14	224/3041 (7.4)		**0.02**
Best				78/1459 (5.4)	Reference	--
Intermediate				119/1377 (8.6)	1.15 (0.85, 1.54)	0.37
Worst				27/205 (13.2)	1.89 (1.20, 2.98)	0.007
**LVMI**	228/3148 (7.2)	1.59 (1.39, 1.82)	**< .0001**	166/2630 (6.3)		**< .0001**
Best				39/1297 (3)	Reference	--
Intermediate				95/1196 (7.9)	1.77 (1.16, 2.69)	0.008
Worst				32/137 (23.4)	4.64 (2.60, 8.26)	< .0001
**Gait Time**	201/2901 (6.9)	1.13 (1.00, 1.28)	**0.04**	135/1989 (6.8)		**0.02**
Best				106/1849 (5.7)	Reference	--
Worst				29/140 (20.7)	1.70 (1.07, 2.70)	0.02
**Grip Strength**	200/2909 (6.9)	0.81 (0.64, 1.03)	0.09	124/1824 (6.8)		0.20
Best				16/332 (4.8)	Reference	--
Intermediate				40/561 (7.1)	0.93 (0.51, 1.71)	0.82
Worst				68/931 (7.3)	1.51 (0.74, 3.06)	0.26

All models are adjusted for age, sex, smoking status, BMI = body mass index (or weight for traits indexed by height), antihypertensive treatment, diabetes status, TC/HDL = ratio of total cholesterol/high-density lipoprotein, and SBP = systolic blood pressure (except when evaluating PP = pulse pressure). Single-occasion trait model hazard ratios (HRs) are reported per standard deviation (SD) increase. Trajectory model HRs are reported for the categorical risk groups.

*Bolded values indicate statistical significance (p<0.05).

CI = Confidence Interval; CRP = C-reactive protein; eGFR = estimated glomerular filtration rate; FEV1 = forced expiratory volume; FVC = forced vital capacity; HbA1c = hemoglobin A1c; HR = heart rate; LVMI = left ventricular mass index.

### Group-based trajectories of traits

**Fig 2:**
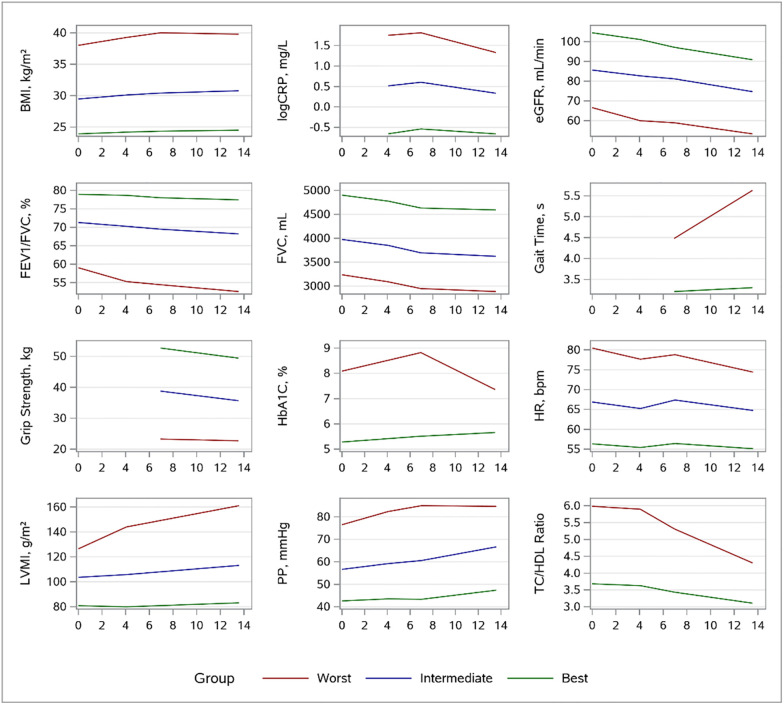
Group-based trajectories for traits. Group-based trajectories for the twelve surrogate traits are shown. For traits resulting in two distinct trajectory profiles, green = ‘best’ and red = ‘worst,’ and if three different profiles emerged, green = ‘best,’ blue = ‘intermediate,’ and red = ‘worst.’ The x-axis represents the number of years since the fifth examination cycle. BMI = body mass index; CRP = C-reactive protein; eGFR = estimated glomerular filtration rate; LVMI = left ventricular mass index; PP = pulse pressure.

The trajectories are shown in Fig 2. For most traits, the trajectory patterns were parallel over time. For LVMI and gait time, the ‘worst’-risk group had a steeper increase while the ‘worst’-risk group trajectory for TC/HDL started relatively high but had a steeper decrease over time. For HbA1c, the ‘worst’-risk group had a non-linear pattern. In exploratory analysis, when excluding participants with prevalent MI and/or previous cardiac surgery (n = 169, 5%) the group-based trajectories remained consistent (S1 Fig and S2 Table). Moreover, the sex-specific exploratory analysis of the single-occasion and group trajectories association in relation to HF risk is shown in S4 and S5 Tables and the corresponding trajectory profiles are in S2 Fig.

### Association of group-based trajectories with HF risk

Adjusting for the covariates noted above, participants with the ‘worst’ trajectory for PP, LVMI, HR, FVC, HbA1c, BMI, CRP, and gait were at higher risk for HF. In contrast to the single-occasion models, we observed a significant positive relation between the trajectory groups for FEV1/FVC and HF risk (Table 2).

The mean change in c-statistic (from a base model) was higher for most traits when using the model with the group-based trajectories than the model with the single-occasion variables (S1 Table).

### Association of trajectory risk score with HF

Based on the forward selection algorithm, the 7 traits selected and used to derive the trajectory risk score were CRP, FVC, gait time, HbA1c, HR, LVMI, and PP. A one-unit increase in the composite trajectory risk score, adjusting for age and sex, was associated with a 2.72-fold increased risk of HF (95% CI [2.21, 3.34]; p<0.001). Moreover, adding the trajectory risk score to a model including age and sex resulted in a higher c-statistic compared to the c-statistic derived from a multiple single-occasion model that included the same 7 single-occasion traits used to derive the risk score, plus age and sex (Table 3).

**Table 3:** C-statistics for models including the trajectory risk score and single-occasion traits.

Models	Variables	C-statistic (95% CI)	Δ2 vs Δ3
Model 1	Age + Sex	0.764 (0.736, 0.791)	
Model 2	Age + Sex + Trajectory Risk Score*	0.867 (0.830, 0.905)	0.103 vs 0.067
Model 3†	Age + Sex + CRP + FVC + Gait time + HbA1c + HR + LVMI + PP	0.831 (0.804, 0.858)	

CRP = C-reactive protein; FVC = forced vital capacity; HbA1c = hemoglobin A1c; HR = heart rate; LVMI = left ventricular mass index; PP = pulse pressure. Bias-corrected c-statistics are reported based on 500 bootstrap samples using the Cox proportional hazards model. Δ2 = mean change in c-statistic between Model 1 and Model 2. Δ3 = mean change in c-statistic between Model 1 and Model 3.

* Based on the forward selection algorithm, the 7 selected traits include CRP, FVC, gait time, HbA1c, HR, LVMI, and PP. The trajectory risk score is derived from the sum of the scores previously assigned to the trajectory variables (1 = ‘best’, 2 = ‘intermediate’, 3 = ‘worst’) and weighted by the corresponding regression coefficients.

^†^ Multiple single-occasion model that includes the same 7 single-occasion traits used to derive the trajectory risk score, plus age and sex.

**Fig 3:**
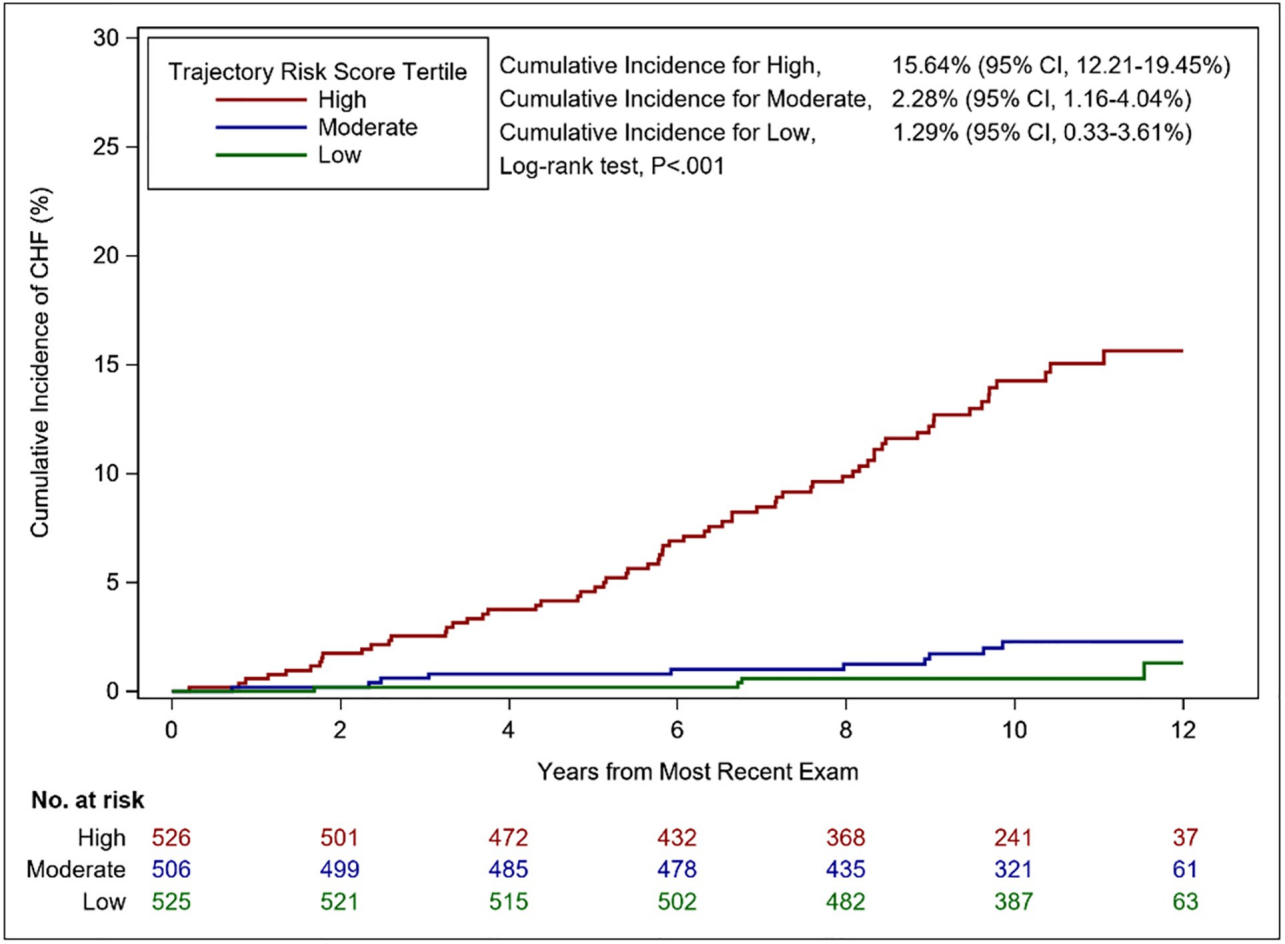
Cumulative incidence of HF by tertiles of the trajectory risk score. The cumulative incidence of heart failure by tertiles of the trajectory risk score is illustrated. Trajectory risk scores are segregated into 3 groups: high = red, moderate = blue, and low = green. The number of participants at risk, every two years since the most recent exam, is included along the x-axis. The median overall follow up for participants with a trajectory risk score was 10.7 years. Log-rank p<0.001.

The cumulative incidence of HF among participants with moderate risk scores (2^nd^ tertile) was 2.3% and among those with high-risk scores (highest tertile) was approximately 16% (Fig 3).

## Discussion

### Principal findings

First, we observed relations of LVMI, PP, CRP, BMI, HbA1c, HR, FVC and gait time with HF risk using either single-occasion measurements or trajectory groups [5–22]. Notably, gait time was positively related to HF risk in both the single-occasion model and the group trajectory models; limited prior data exist on this relation among people free of HF. Second, we did not observe a relation between grip strength and HF risk using either model. Third, we observed a positive relation between the FEV1/FVC and HF risk using the trajectory model, but not as a single occasion measurement. Fourth, we observed positive relations of TC/HDL and eGFR with HF risk when the single-occasion model was used, but not with trajectory groups. Fifth, using the forward selection algorithm, seven traits were derived to create the trajectory risk score: CRP, FVC, gait time, HbA1c, HR, LVMI, and PP. Sixth, we observed that a one-unit increase in the composite multi-system trajectory risk score was associated with an approximately three-fold increase in HF risk adjusting for standard HF risk factors. Seventh, adding the trajectory risk score to a model including age and sex resulted in a higher increase in the c-statistic compared to the addition of single-occasion variables to an age-, and sex-adjusted model. Finally, participants with values in the highest tertile of the trajectory score had an absolute HF risk that was substantially elevated compared to the lower two tertiles.

### Comparison with the literature

Prior studies have demonstrated a link between the aging of the population and an increased incidence of HF [3, 5]. However, it has become apparent that not all aging is the same [25–27]. Most prior reports have focused on the relations between traits measured on a single occasion and HF risk [4–24]. Our investigation demonstrated that most single-occasion traits are weakly correlated with each other, indicating that the function of individual organ systems may follow different trajectories during the aging process.

Previous studies have reported on the relations of single-occasion traits including TC/HDL, PP, HR, CRP, BMI, LVMI, and HbA1c, with HF risk, consistent with our study findings [5–16]; this supports the established associations of a pro-inflammatory state (CRP), sympathetic hyperactivity (HR), coronary heart disease (TC/HDL, PP, BMI, LVMI, HbA1c), with the risk of incident HF. We also confirmed an inverse relation of FVC with HF risk [19, 20]. The association of lung dysfunction with HF risk has been attributed to altered pre-load and after-load conditions on the heart, an inflammatory state, and increased oxidative stress on the myocardium. Furthermore, our findings supported the previously noted inverse relation of eGFR with HF risk [17, 18]. Kidney dysfunction has been associated with an increased risk of HF due to an altered renin-angiotensin-aldosterone system which is integral to the body’s management of blood volume and blood pressure [18]. Although grip strength has been linked to cardiovascular fitness and risk of HF, we did not observe an association between grip strength and incident HF; prior data for this trait are limited, but two previous investigations reported an inverse relation [23, 24]. Gait time has been associated with mortality, cardiovascular fitness, and recurrent HF in individuals with prevalent HF; we extended these findings by confirming a direct association between gait time and future HF risk in individuals without HF, which, to our knowledge, had only been reported in one previous study [22, 33, 34].

Given that organ systems have been shown to follow different aging patterns, we created trajectories of the selected traits to provide additional insight into future disease risk [25–27, 32]. To our knowledge, the association of organ system trajectories to incident HF has not been previously investigated. In our analysis, we leveraged serial trait measurements to create trajectory groups that were associated with a higher risk of HF. In contrast to the single-occasion trait model that did not show an association between FEV1/FVC and incident HF, the ‘worst’ subgroup trajectory for FEV1/FVC was associated with higher HF risk. Based on our results, lung dysfunction may only be clinically relevant to HF risk in the subgroup of individuals with the most drastic decline in lung function over time [21]. Similar to the single occasion trait model, we did not observe an association between grip strength trajectory and incident HF, indicating that the trajectory for grip strength may not be an ideal marker for future HF risk. Additionally, the ‘worst’ TC/HDL trajectory did not show a significant relation to incident HF possibly because there was improvement in the participants’ cholesterol profiles over time. Lastly, we did not observe an association of the eGFR trajectory with HF risk, suggesting that the most recent measurement of eGFR may be a better indicator of future HF risk than its trajectory.

Our investigation demonstrated that individuals with suboptimal trajectory scores across multiple systems were at the highest risk of HF. The incremental discrimination ability of models including trajectory variables (above and beyond that of a model including age and sex) was greater than the ability of a model including single-occasion traits. Furthermore, we observed a positive association between the trajectory score and HF risk. Our results suggest that trajectories may provide a more powerful tool in discriminating the most at-risk individuals for future HF. Ultimately, we hope that trajectory scores may provide clinicians with better insight into ascertaining which individuals are at highest risk of future HF. This may allow for earlier intervention and targeted therapy that would hopefully alter the disease course leading to clinical HF. Trajectory scores may provide clinicians with a more comprehensive understanding of a person’s risk of future HF by incorporating the aging of multiple organ systems over time into one model. Future studies are needed to assess whether incorporating a trajectory score into an individual’s assessment alters disease management and reduces risk of future HF.

## Study strengths and limitations

The participants of this investigation were from a large, community-based cohort, followed over a prolonged period. We chose 12 traits to provide a comprehensive representation of the body’s functions and metabolism. There are several limitations of this investigation. First, there was a potential for survival bias given that participants who attended the fifth examination cycle and at least one other examination cycle after 1995 were eligible for inclusion. Second, the participants consisted mainly of white adults of European ancestry, limiting the generalizability of our findings to other races and ethnicities. Third, this was a prospective cohort investigation, and therefore, cause and effect cannot be inferred from the associations we observed.

## Conclusion

Our findings support the incremental predictive value of long-term repeated measurements of multiple organ systems in evaluating HF risk, which may ultimately provide better insight into ascertaining which individuals are at greatest risk of future HF. Additional studies are needed to determine whether earlier interventions targeted towards individuals with poor trajectory risk profiles may prevent future HF.

## Supporting information

S1 Fig Group trajectories for the surrogate traits. (DOCX)

S2 Fig Sex-specific group trajectories for surrogate traits. (DOCX)

S1 Table C-statistics for single trait and group-based trajectory models. Bias-corrected c-statistics are reported based on 500 bootstrap samples using the Cox proportional hazards model. Model 0 includes the covariates: age, sex, smoking status, BMI = body mass index (or weight for traits indexed by height), antihypertensive treatment, diabetes status, TC/HDL = total cholesterol/high-density lipoprotein, and systolic blood pressure (except when evaluating pulse pressure). Model 1 includes the covariates plus the single occasion variable. Model 2 includes the covariates plus the group-based trajectory variable. ΔS_1_ = mean change in c-statistic between Model 0 and Model 1. ΔS_2_ = mean change in c-statistic between Model 0 and Model 2. CRP = C-reactive protein; eGFR = estimated glomerular filtration rate; FEV1 = forced expiratory volume; FVC = forced vital capacity; HbA1c = hemoglobin A1c; HR = heart rate; LVMI = left ventricular mass index.
(DOCX)

S2 Table Associations of single-occasion traits and group trajectories with HF risk (excluding prevalent MI and/or previous cardiac surgery). (DOCX)

S3 Table Number of measurements by trait. (DOCX)

S4 Table Associations of single-occasion traits and group trajectories with HF risk for men. (DOCX)

S5 Table Associations of single-occasion traits and group trajectories with HF risk for women. (DOCX)
