# Supplementary material for: Multi-system trajectories and the incidence of heart failure in the Framingham Offspring Study
Source: PLoS One. 2022 May 26;17(5):e0268576. doi: 10.1371/journal.pone.0268576 (PMC9135195; doi:10.1371/journal.pone.0268576)
Supplement: S2 Fig — (DOCX) [file pone.0268576.s002.docx]

**S2 Fig.** Sex-Specific Group-Based Trajectories for Traits


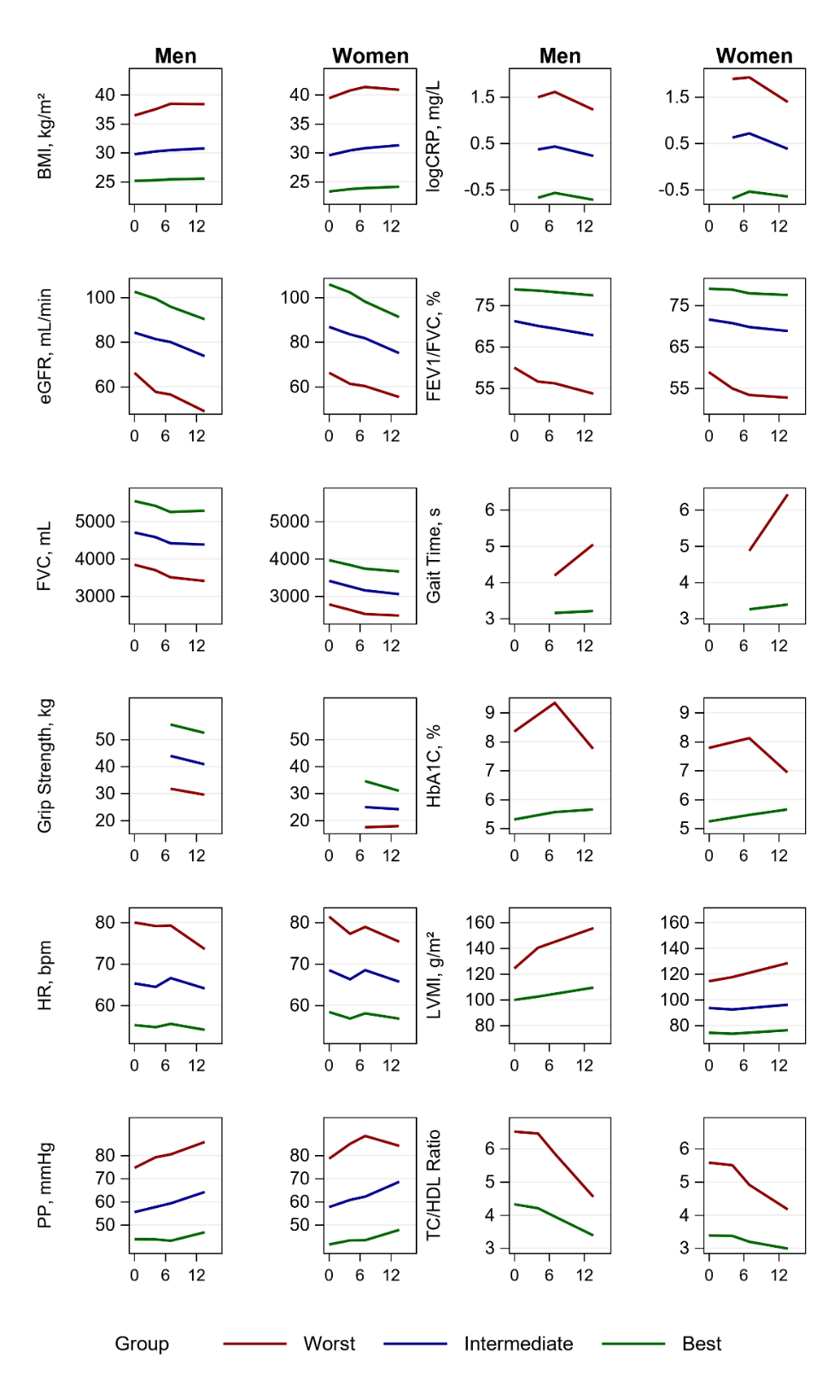


Group-based trajectories for the twelve surrogate traits are shown. For traits resulting in two distinct trajectory profiles, green=‘best’ and red=‘worst,’ and if three different profiles emerged, green=‘best,’ blue=‘intermediate,’ and red=‘worst.’ The x-axis represents the number of years since the fifth examination cycle.

BMI=body mass index; CRP=C-reactive protein; eGFR=estimated glomerular filtration rate; LVMI=left ventricular mass index; PP=pulse pressure.
