# Supplementary material for: Multi-system trajectories and the incidence of heart failure in the Framingham Offspring Study
Source: PLoS One. 2022 May 26;17(5):e0268576. doi: 10.1371/journal.pone.0268576 (PMC9135195; doi:10.1371/journal.pone.0268576)
Supplement: S1 Table — Bias-corrected c-statistics are reported based on 500 bootstrap samples using the Cox proportional hazards model. Model 0 includes the covariates: age, sex, smoking status, BMI = body mass index (or weight for traits indexed by height), antihypertensive treatment, diabetes status, TC/HDL = total cholesterol/high-density lipoprotein, and systolic blood pressure (except when evaluating pulse pressure). Model 1 includes the covariates plus the single occasion variable. Model 2 includes the covariates plus the group-based trajectory variable. ΔS1 = mean change in c-statistic between Model 0 and Model 1. ΔS2 = mean change in c-statistic between Model 0 and Model 2. CRP = C-reactive protein; eGFR = estimated glomerular filtration rate; FEV1 = forced expiratory volume; FVC = forced vital capacity; HbA1c = hemoglobin A1c; HR = heart rate; LVMI = left ventricular mass index. (DOCX) [file pone.0268576.s003.docx]

**S1 Table**. C-statistics for Single Trait and Group-Based Trajectory Models

| Trait | **Model 0 (S_0_)**  C-statistic (95% CI) | **Model 1 (S_1_)**  C-statistic (95% CI) | **Δ S_1_** | **Model 2 (S_2_)**  C-statistic (95% CI) | **Δ S_2_** | **Δ S_2_ vs Δ S_1_** |
| --- | --- | --- | --- | --- | --- | --- |
| eGFR | 0.804 (0.780, 0.827) | 0.803 (0.780, 0.827) | -0.0002 | 0.808 (0.785, 0.832) | 0.005 | 0.005 vs -0.0002 |
| HbA1c | 0.804 (0.780, 0.827) | 0.812 (0.788, 0.837) | 0.009 | 0.811 (0.786, 0.836) | 0.007 | 0.007 vs 0.009 |
| BMI | 0.796 (0.773, 0.819) | 0.804 (0.780, 0.827) | 0.008 | 0.802 (0.778, 0.825) | 0.006 | 0.006 vs 0.008 |
| Pulse Pressure | 0.803 (0.780, 0.827) | 0.804 (0.781, 0.827) | 0.001 | 0.807 (0.784, 0.831) | 0.004 | 0.004 vs 0.001 |
| CRP | 0.804 (0.780, 0.827) | 0.811 (0.788, 0.835) | 0.008 | 0.814 (0.789, 0.838) | 0.010 | 0.010 vs 0.008 |
| Heart Rate | 0.804 (0.780, 0.827) | 0.807 (0.784, 0.830) | 0.003 | 0.809 (0.786, 0.832) | 0.006 | 0.006 vs 0.003 |
| TC/HDL Ratio | 0.792 (0.768, 0.815) | 0.795 (0.771, 0.818) | 0.003 | 0.801 (0.777, 0.825) | 0.010 | 0.010 vs 0.003 |
| FVC | 0.804 (0.780, 0.827) | 0.816 (0.791, 0.840) | 0.012 | 0.821 (0.797, 0.844) | 0.017 | 0.017 vs 0.012 |
| FEV1/FVC | 0.804 (0.780, 0.827) | 0.806 (0.782, 0.831) | 0.003 | 0.811 (0.785, 0.838) | 0.008 | 0.008 vs 0.003 |
| LVMI | 0.800 (0.777, 0.823) | 0.824 (0.800, 0.847) | 0.023 | 0.829 (0.800, 0.858) | 0.029 | 0.029 vs 0.023 |
| Gait Time | 0.804 (0.780, 0.827) | 0.810 (0.785, 0.836) | 0.007 | 0.828 (0.798, 0.858) | 0.025 | 0.025 vs 0.007 |
| Grip Strength | 0.804 (0.780, 0.827) | 0.807 (0.781, 0.834) | 0.004 | 0.833 (0.803, 0.864) | 0.030 | 0.030 vs 0.004 |

Bias-corrected c-statistics are reported based on 500 bootstrap samples using the Cox proportional hazards model. Model 0 includes the covariates: age, sex, smoking status, BMI=body mass index (or weight for traits indexed by height), antihypertensive treatment, diabetes status, TC/HDL=total cholesterol/high-density lipoprotein, and systolic blood pressure (except when evaluating pulse pressure). Model 1 includes the covariates plus the single occasion variable. Model 2 includes the covariates plus the group-based trajectory variable. ΔS_1_=mean change in c-statistic between Model 0 and Model 1. ΔS_2_=mean change in c-statistic between Model 0 and Model 2.

CRP=C-reactive protein; eGFR=estimated glomerular filtration rate; FEV1=forced expiratory volume; FVC=forced vital capacity; HbA1c=hemoglobin A1c; HR=heart rate; LVMI=left ventricular mass index.
