# Supplementary material for: Multi-system trajectories and the incidence of heart failure in the Framingham Offspring Study
Source: PLoS One. 2022 May 26;17(5):e0268576. doi: 10.1371/journal.pone.0268576 (PMC9135195; doi:10.1371/journal.pone.0268576)
Supplement: S2 Table — (DOCX) [file pone.0268576.s004.docx]

**S2 Table**. Associations of Single-Occasion Traits and Group Trajectories with HF risk (excluding prevalent MI and/or previous cardiac surgery)

| **Trait** | **Model 1 – Single Occasion** | | | **Model 2 – Group Trajectory** | | |
| --- | --- | --- | --- | --- | --- | --- |
|  | **# events/# at risk (%)** | **HR (95% CI)** | **p-value*** | **# events/# at risk (%)** | **HR (95% CI)** | **p-value*** |
| **eGFR** | 235/3234 (7.3) | 0.89 (0.77, 1.03) | 0.11 | 229/3156 (7.3) |  | 0.08 |
| Best |  |  |  | 29/1038 (2.8) | Reference | -- |
| Intermediate |  |  |  | 118/1625 (7.3) | 1.00 (0.63, 1.57) | 0.99 |
| Worst |  |  |  | 82/493 (16.6) | 1.42 (0.84, 2.40) | 0.19 |
| **HbA1c** | 225/3157 (7.1) | 1.31 (1.17, 1.47) | **<.0001** | 180/2672 (6.7) |  | **0.02** |
| Best |  |  |  | 163/2578 (6.3) | Reference | -- |
| Worst |  |  |  | 17/94 (18.1) | 1.96 (1.11, 3.48) | 0.02 |
| **BMI** | 235/3243 (7.2) | 1.39 (1.23, 1.57) | **<.0001** | 234/3199 (7.3) |  | **<.0001** |
| Best |  |  |  | 85/1608 (5.3) | Reference | -- |
| Intermediate |  |  |  | 111/1329 (8.4) | 1.28 (0.96, 1.72) | 0.10 |
| Worst |  |  |  | 38/262 (14.5) | 2.76 (1.82, 4.19) | <.0001 |
| **PP** | 235/3243 (7.2) | 1.20 (1.04, 1.38) | **0.01** | 235/3243 (7.3) |  | **<.0001** |
| Best |  |  |  | 37/1666 (2.2) | Reference | -- |
| Intermediate |  |  |  | 118/1182 (10) | 2.05 (1.35, 3.13) | 0.001 |
| Worst |  |  |  | 80/395 (20.3) | 3.24 (1.98, 5.33) | <.0001 |
| **CRP** | 233/3190 (7.3) | 1.29 (1.15, 1.44) | **<.0001** | 202/2916 (6.9) |  | **0.001** |
| Best |  |  |  | 11/532 (2.1) | Reference | --- |
| Intermediate |  |  |  | 87/1403 (6.2) | 1.81 (0.96, 3.42) | 0.06 |
| Worst |  |  |  | 104/981 (10.6) | 2.80 (1.47, 5.34) | 0.002 |
| **HR** | 235/3243 (7.2) | 1.26 (1.12, 1.43) | **0.0002** | 235/3243 (7.3) |  | **0.0005** |
| Best |  |  |  | 47/1056 (4.5) | Reference | -- |
| Intermediate |  |  |  | 131/1754 (7.5) | 1.54 (1.09, 2.16) | 0.01 |
| Worst |  |  |  | 57/433 (13.2) | 2.27 (1.50, 3.41) | <.0001 |
| **TC/HDL** | 235/3243 (7.2) | 1.12 (1.03, 1.21) | **0.005** | 233/3190 (7.3) |  | 0.93 |
| Best |  |  |  | 145/2146 (6.8) | Reference | -- |
| Worst |  |  |  | 88/1044 (8.4) | 1.01 (0.77, 1.34) | 0.93 |
| **FVC** | 203/2990 (6.8) | 0.59 (0.47, 0.75) | **<.0001** | 194/2906 (6.7) |  | **0.002** |
| Best |  |  |  | 12/608 (2) | Reference | -- |
| Intermediate |  |  |  | 72/1437 (5) | 1.71 (0.92, 3.17) | 0.09 |
| Worst |  |  |  | 110/861 (12.8) | 2.59 (1.39, 4.84) | 0.003 |
| **FEV1/FVC** | 222/3143 (7.1) | 0.89 (0.78, 1.02) | 0.09 | 194/2906 (6.7) |  | 0.11 |
| Best |  |  |  | 69/1384 (5) | Reference | -- |
| Intermediate |  |  |  | 99/1310 (7.6) | 1.03 (0.75, 1.42) | 0.84 |
| Worst |  |  |  | 26/212 (12.3) | 1.60 (1.00, 2.55) | 0.049 |
| **LVMI** | 197/3006 (6.6) | 1.58 (1.37, 1.83) | **<.0001** | 140/2529 (5.5) |  | **<.0001** |
| Best |  |  |  | 36/1266 (2.8) | Reference | -- |
| Intermediate |  |  |  | 81/1149 (7.1) | 1.76 (1.14, 2.74) | 0.01 |
| Worst |  |  |  | 23/114 (20.2) | 4.44 (2.33, 8.46) | <.0001 |
| **Gait Time** | 177/2781 (6.4) | 1.13 (1.00, 1.29) | 0.06 | 116/1924 (6) |  | 0.06 |
| Best |  |  |  | 93/1802 (5.2) | Reference | -- |
| Worst |  |  |  | 23/122 (18.9) | 1.61 (0.97, 2.67) | 0.06 |
| **Grip Strength** | 176/2787 (6.3) | 0.83 (0.64, 1.08) | 0.17 | 105/1763 (6) |  | 0.13 |
| Best |  |  |  | 15/315 (4.8) | Reference | -- |
| Intermediate |  |  |  | 31/543 (5.7) | 0.69 (0.36, 1.31) | 0.25 |
| Worst |  |  |  | 59/905 (6.5) | 1.20 (0.56, 2.57) | 0.64 |

All models are adjusted for age, sex, smoking status, BMI=body mass index (or weight for traits indexed by height), antihypertensive treatment, diabetes status, TC/HDL=ratio of total cholesterol/high-density lipoprotein, and SBP=systolic blood pressure (except when evaluating PP=pulse pressure). Single-occasion trait model hazard ratios (HRs) are reported per standard deviation (SD) increase. Trajectory model HRs are reported for the categorical risk groups. ***Bolded** values indicate statistical significance (p<0.05).

CI=Confidence Interval; CRP=C-reactive protein; eGFR=estimated glomerular filtration rate; FEV1=forced expiratory volume; FVC=forced vital capacity; HbA1c=hemoglobin A1c; HR=heart rate; LVMI=left ventricular mass index.
