# Supplementary material for: Multi-system trajectories and the incidence of heart failure in the Framingham Offspring Study
Source: PLoS One. 2022 May 26;17(5):e0268576. doi: 10.1371/journal.pone.0268576 (PMC9135195; doi:10.1371/journal.pone.0268576)
Supplement: S3 Table — (DOCX) [file pone.0268576.s005.docx]

**S3 Table.** Number of Measurements by Trait

| Trait | Exam Cycles of Measurement Availability | Maximum Number of Measurements Possible per Participant (%) | Average Number of Measurements per Participant (SD) |
| --- | --- | --- | --- |
| eGFR | 5, 6, 7, 8 | 4.0 (60%) | 3.4 (0.8) |
| HbA1c | 5, 7, 8 | 3.0 (50%) | 2.3 (0.8) |
| BMI | 5, 6, 7, 8 | 4.0 (72%) | 3.6 (0.7) |
| PP | 5, 6, 7, 8 | 4.0 (75%) | 3.7 (0.6) |
| CRP | 6, 7, 8 | 3.0 (69%) | 2.6 (0.7) |
| HR | 5, 6, 7, 8 | 4.0 (75%) | 3.7 (0.6) |
| TC/HDL | 5, 6, 7, 8 | 4.0 (71%) | 3.6 (0.7) |
| FVC | 5, 6, 7, 8 | 4.0 (43%) | 3.0 (1.1) |
| FEV1/FVC | 5, 6, 7, 8 | 4.0 (43%) | 3.0 (1.1) |
| LVMI | 5, 6, 8 | 3.0 (48%) | 2.2 (1.0) |
| Gait Time | 7, 8 | 2.0 (58%) | 1.4 (0.7) |
| Grip Strength | 7, 8 | 2.0 (53%) | 1.4 (0.7) |
