# Supplementary material for: Multi-system trajectories and the incidence of heart failure in the Framingham Offspring Study
Source: PLoS One. 2022 May 26;17(5):e0268576. doi: 10.1371/journal.pone.0268576 (PMC9135195; doi:10.1371/journal.pone.0268576)
Supplement: S4 Table — (DOCX) [file pone.0268576.s006.docx]

**S4 Table.** Associations of Single-Occasion Traits and Group Trajectories with HF risk for Men

| **Men** | | | | | | |
| --- | --- | --- | --- | --- | --- | --- |
| **Trait** | **Model 1 – Single Occasion** | | | **Model 2 – Group Trajectory** | | |
|  | **# events/# at risk (%)** | **HR (95% CI)** | **p-value*** | **# events/# at risk (%)** | **HR (95% CI)** | **p-value*** |
| **eGFR** | 138/1569 (8.8) | 0.77 (0.64, 0.92) | **0.004** | 134/1536 (8.7) |  | **0.02** |
| Best |  |  |  | 21/578 (3.6) | Reference | -- |
| Intermediate |  |  |  | 77/771 (10) | 1.47 (0.85, 2.56) | 0.17 |
| Worst |  |  |  | 36/187 (19.3) | 2.40 (1.23, 4.71) | 0.01 |
| **HbA1c** | 134/1527 (8.8) | 1.23 (1.02, 1.47) | **0.03** | 106/1263 (8.4) |  | 0.32 |
| Best |  |  |  | 97/1222 (7.9) | Reference | -- |
| Worst |  |  |  | 9/41 (22) | 1.47 (0.69, 3.12) | 0.32 |
| **BMI** | 140/1573 (8.9) | 1.43 (1.21, 1.70) | **<.0001** | 137/1554 (8.8) |  | **0.0004** |
| Best |  |  |  | 49/756 (6.5) | Reference | -- |
| Intermediate |  |  |  | 69/656 (10.5) | 1.59 (1.09, 2.32) | 0.02 |
| Worst |  |  |  | 19/142 (13.4) | 3.09 (1.75, 5.46) | <.0001 |
| **PP** | 140/1573 (8.9) | 1.04 (0.86, 1.26) | 0.68 | 140/1573 (8.9) |  | 0.12 |
| Best |  |  |  | 33/775 (4.3) | Reference | -- |
| Intermediate |  |  |  | 72/604 (11.9) | 1.55 (0.97, 2.48) | 0.07 |
| Worst |  |  |  | 35/194 (18) | 1.80 (0.99, 3.27) | 0.05 |
| **CRP** | 137/1551 (8.8) | 1.29 (1.11, 1.49) | **0.0006** | 120/1403 (8.6) |  | **0.0002** |
| Best |  |  |  | 3/223 (1.4) | Reference | --- |
| Intermediate |  |  |  | 44/692 (6.4) | 3.01 (0.93, 9.79) | 0.07 |
| Worst |  |  |  | 73/488 (15) | 5.91 (1.81, 19.30) | 0.003 |
| **HR** | 140/1573 (8.9) | 1.18 (1.01, 1.38) | **0.04** | 140/1573 (8.9) |  | **0.003** |
| Best |  |  |  | 39/619 (6.3) | Reference | -- |
| Intermediate |  |  |  | 70/771 (9.1) | 1.33 (0.89, 1.97) | 0.16 |
| Worst |  |  |  | 31/183 (16.9) | 2.37 (1.44, 3.91) | 0.0007 |
| **TC/HDL** | 140/1573 (8.9) | 1.14 (1.02, 1.27) | **0.02** | 137/1552 (8.8) |  | 0.85 |
| Best |  |  |  | 94/1102 (8.5) | Reference | -- |
| Worst |  |  |  | 43/450 (9.6) | 1.04 (0.72, 1.50) | 0.85 |
| **FVC** | 119/1444 (8.2) | 0.66 (0.52, 0.84) | **0.0008** | 109/1397 (7.8) |  | **0.02** |
| Best |  |  |  | 3/332 (0.9) | Reference | -- |
| Intermediate |  |  |  | 48/677 (7.1) | 4.28 (1.32, 13.92) | 0.02 |
| Worst |  |  |  | 58/388 (15) | 5.41 (1.61, 18.13) | 0.006 |
| **FEV1/FVC** | 133/1534 (8.7) | 0.99 (0.82, 1.20) | 0.94 | 109/1397 (7.8) |  | 0.41 |
| Best |  |  |  | 33/612 (5.4) | Reference | -- |
| Intermediate |  |  |  | 64/661 (9.7) | 1.33 (0.87, 2.05) | 0.19 |
| Worst |  |  |  | 12/124 (9.7) | 1.33 (0.67, 2.64) | 0.41 |
| **LVMI** | 114/1415 (8.1) | 1.67 (1.40, 1.98) | **<.0001** | 80/1138 (7) |  | **<.0001** |
| Best |  |  |  | 45/984 (4.6) | Reference | -- |
| Worst |  |  |  | 35/154 (22.7) | 3.25 (1.99, 5.31) | <.0001 |
| **Gait Time** | 106/1327 (8) | 1.04 (0.86, 1.25) | 0.68 | 80/898 (8.9) |  | **0.006** |
| Best |  |  |  | 56/807 (6.9) | Reference | -- |
| Worst |  |  |  | 24/91 (26.4) | 2.15 (1.25, 3.71) | 0.006 |
| **Grip Strength** | 105/1329 (7.9) | 0.79 (0.61, 1.01) | 0.06 | 73/822 (8.9) |  | 0.13 |
| Best |  |  |  | 7/183 (3.8) | Reference | -- |
| Intermediate |  |  |  | 33/468 (7.1) | 0.85 (0.36, 2.01) | 0.71 |
| Worst |  |  |  | 33/171 (19.3) | 1.46 (0.57, 3.70) | 0.43 |
