# Supplementary material for: Multi-system trajectories and the incidence of heart failure in the Framingham Offspring Study
Source: PLoS One. 2022 May 26;17(5):e0268576. doi: 10.1371/journal.pone.0268576 (PMC9135195; doi:10.1371/journal.pone.0268576)
Supplement: S5 Table — (DOCX) [file pone.0268576.s007.docx]

**S5 Table.** Associations of Single-Occasion Traits and Group Trajectories with HF risk for Women

| **Women** | | | | | | |
| --- | --- | --- | --- | --- | --- | --- |
| **Trait** | **Model 1 – Single Occasion** | | | **Model 2 – Group Trajectory** | | |
|  | **# events/# at risk (%)** | **HR (95% CI)** | **p-value*** | **# events/# at risk (%)** | **HR (95% CI)** | **p-value*** |
| **eGFR** | 136/1832 (7.4) | 1.02 (0.85, 1.24) | 0.81 | 128/1776 (7.2) |  | 0.60 |
| Best |  |  |  | 16/553 (2.9) | Reference | -- |
| Intermediate |  |  |  | 65/923 (7) | 0.73 (0.40, 1.35) | 0.32 |
| Worst |  |  |  | 47/300 (15.7) | 0.78 (0.39, 1.59) | 0.50 |
| **HbA1c** | 128/1792 (7.1) | 1.23 (1.08, 1.39) | **0.002** | 99/1531 (6.5) |  | 0.06 |
| Best |  |  |  | 86/1464 (5.9) | Reference | -- |
| Worst |  |  |  | 13/67 (19.4) | 1.92 (0.97, 3.78) | 0.06 |
| **BMI** | 136/1839 (7.4) | 1.27 (1.07, 1.51) | **0.006** | 133/1805 (7.4) |  | **<.0001** |
| Best |  |  |  | 57/1044 (5.5) | Reference | -- |
| Intermediate |  |  |  | 52/623 (8.4) | 1.09 (0.74, 1.62) | 0.66 |
| Worst |  |  |  | 24/138 (17.4) | 2.17 (1.27, 3.70) | 0.005 |
| **PP** | 136/1839 (7.4) | 1.25 (1.05, 1.49) | **0.01** | 136/1839 (7.4) |  | **0.02** |
| Best |  |  |  | 19/977 (1.9) | Reference | -- |
| Intermediate |  |  |  | 74/665 (11.1) | 2.06 (1.14, 3.72) | 0.02 |
| Worst |  |  |  | 43/197 (21.8) | 2.79 (1.39, 5.60) | 0.004 |
| **CRP** | 132/1799 (7.3) | 1.22 (1.03, 1.43) | **0.02** | 109/1649 (6.6) |  | 0.67 |
| Best |  |  |  | 9/318 (2.8) | Reference | --- |
| Intermediate |  |  |  | 50/757 (6.6) | 1.37 (0.67, 2.81) | 0.39 |
| Worst |  |  |  | 50/574 (8.7) | 1.37 (0.65, 2.92) | 0.40 |
| **HR** | 136/1839 (7.4) | 1.23 (1.04, 1.46) | **0.01** | 136/1839 (7.4) |  | **0.004** |
| Best |  |  |  | 17/571 (3) | Reference | -- |
| Intermediate |  |  |  | 83/1017 (8.2) | 2.00 (1.18, 3.40) | 0.01 |
| Worst |  |  |  | 36/251 (14.3) | 2.73 (1.51, 4.95) | 0.001 |
| **TC/HDL** | 136/1839 (7.4) | 1.21 (1.02, 1.43) | **0.03** | 132/1798 (7.3) |  | 0.63 |
| Best |  |  |  | 85/1322 (6.4) | Reference | -- |
| Worst |  |  |  | 47/476 (9.9) | 1.10 (0.76, 1.58) | 0.63 |
| **FVC** | 117/1689 (6.9) | 0.57 (0.44, 0.73) | **<.0001** | 115/1644 (7) |  | **<.0001** |
| Best |  |  |  | 4/350 (1.1) | Reference | -- |
| Intermediate |  |  |  | 34/813 (4.2) | 2.21 (0.78, 6.26) | 0.13 |
| Worst |  |  |  | 77/481 (16) | 5.07 (1.82, 14.16) | 0.002 |
| **FEV1/FVC** | 126/1765 (7.1) | 0.82 (0.70, 0.98) | **0.03** | 115/1644 (7) |  | 0.08 |
| Best |  |  |  | 44/829 (5.3) | Reference | -- |
| Intermediate |  |  |  | 55/714 (7.7) | 0.99 (0.66, 1.50) | 0.97 |
| Worst |  |  |  | 16/101 (15.8) | 1.85 (1.02, 3.35) | 0.04 |
| **LVMI** | 114/1733 (6.6) | 1.36 (1.14, 1.61) | **0.0005** | 86/1492 (5.8) |  | **0.02** |
| Best |  |  |  | 16/695 (2.3) | Reference | -- |
| Intermediate |  |  |  | 47/681 (6.9) | 1.79 (0.98, 3.27) | 0.06 |
| Worst |  |  |  | 23/116 (19.8) | 2.77 (1.33, 5.76) | 0.007 |
| **Gait Time** | 95/1574 (6) | 1.26 (1.07, 1.48) | **0.005** | 55/1091 (5) |  | 0.34 |
| Best |  |  |  | 48/1044 (4.6) | Reference | -- |
| Worst |  |  |  | 7/47 (14.9) | 1.54 (0.63, 3.75) | 0.34 |
| **Grip Strength** | 95/1580 (6) | 1.00 (0.79, 1.26) | 0.99 | 51/1002 (5.1) |  | 0.77 |
| Best |  |  |  | 2/174 (1.2) | Reference | -- |
| Intermediate |  |  |  | 22/579 (3.8) | 1.52 (0.34, 6.85) | 0.59 |
| Worst |  |  |  | 27/249 (10.8) | 1.75 (0.36, 8.59) | 0.49 |

All models are adjusted for age, sex, smoking status, BMI=body mass index (or weight for traits indexed by height), antihypertensive treatment, diabetes status, TC/HDL=ratio of total cholesterol/high-density lipoprotein, and SBP=systolic blood pressure (except when evaluating PP=pulse pressure). Single-occasion trait model hazard ratios (HRs) are reported per standard deviation (SD) increase. Trajectory model HRs are reported for the categorical risk groups. ***Bolded** values indicate statistical significance (p<0.05).

CI=Confidence Interval; CRP=C-reactive protein; eGFR=estimated glomerular filtration rate; FEV1=forced expiratory volume; FVC=forced vital capacity; HbA1c=hemoglobin A1c; HR=heart rate; LVMI=left ventricular mass index.
